# Association between dietary creatine intake and serum biomarkers of spermatogenesis in males aged 12 years and older

**DOI:** 10.1530/RAF-25-0009

**Published:** 2025-09-03

**Authors:** David Nedeljkovic, Nikola Todorovic, Tonje Holte Stea, Dagrun Engeset, Sergej M Ostojic

**Affiliations:** ^1^ Applied Bioenergetics Lab, Faculty of Sport and Physical Education, University of Novi Sad, Novi Sad, Serbia; ^2^ Department of Health and Nursing Sciences, University of Agder, Kristiansand, Norway; ^3^ Department of Nutrition and Public Health, University of Agder, Kristiansand, Norway; ^4^ Faculty of Health Sciences, University of Pécs, Pécs, Hungary

**Keywords:** creatine, spermatogenesis, inhibin B

## Abstract

**Graphical Abstract:**

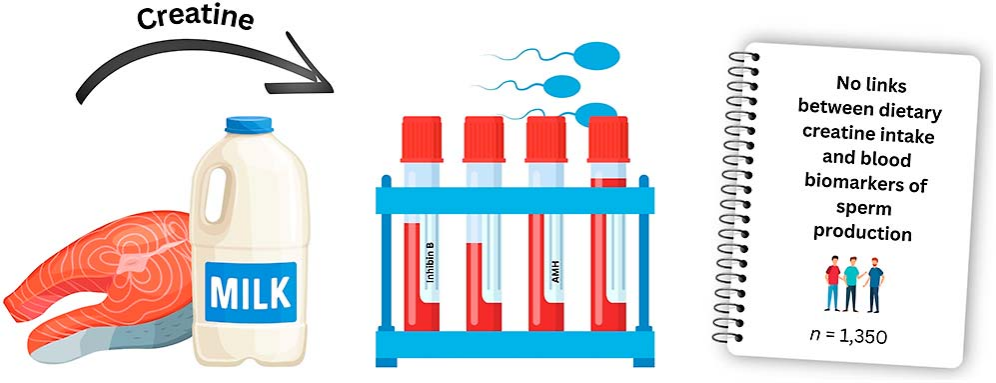

**Lay summary:**

Creatine is a nutrient that helps provide energy to different parts of the body, including the testes. Since making sperm takes a lot of energy, creatine might play a role in supporting sperm health. Some studies suggest it could help, while others raise concerns. However, it is still unclear what effect, if any, creatine from food has on sperm. To learn more, we looked at data from over 1,300 males in the US, aged 12 and older. We compared how much creatine they got from their diet with levels of two hormones in the blood that are related to sperm production. We found no strong link between dietary creatine and these hormone levels. This suggests that creatine from food probably does not have a major effect – good or bad – on male reproductive health. Since this study used existing data and did not test creatine directly in an experiment, we cannot say for sure if creatine causes any changes. More research, especially studies that look directly at sperm, is needed to better understand how creatine might affect fertility.

Creatine is a conditionally essential nutrient that plays a vital role in replenishing energy in various metabolically demanding organs, including the testes. Spermatogenesis is a highly energy-intensive process, with creatine facilitating energy transfer to support sperm cell survival and resilience against environmental stress (for a comprehensive review, see Ostojic *et al.* (2022)). Several preliminary studies have reported beneficial effects of exogenous creatine or creatine precursors on sperm viability (Tapeh *et al.* 2017, Kuribayashi *et al.* 2024). However, a few preclinical studies and media reports have suggested potential spermatotoxic effects of creatine (Cap Score 2024), creating uncertainty regarding its role in male fertility. Notably, the association between dietary creatine intake and biomarkers of spermatogenesis in humans remains largely unexplored, particularly at a population level. To address this gap, we examined the relationship between dietary creatine intake and serum biomarkers of spermatogenesis in US males aged 12 years and older, utilizing data from the National Health and Nutrition Examination Survey (NHANES).

For this report, we analyzed data from the NHANES cycles 1999–2004, focusing on male participants aged 12 years and older who provided detailed dietary intake information and had serum levels of inhibin B and anti-Müllerian hormone (AMH) measured using enzyme-linked immunoassays. Daily creatine intake (grams per day) was calculated based on the average creatine content of various food sources, including 0.20 g/kg for milk-based foods and 3.88 g/kg for meat-based sources. The total intake was determined by summing the creatine contributions from all creatine-containing foods consumed by each participant. This calculation exclusively considered creatine obtained from dietary sources and did not include creatine from dietary supplements or pharmacological agents. The dataset was curated to ensure completeness, and only participants with available data on both dietary intake and serum biomarkers of spermatogenesis were included in the final analysis.

A total of 1,350 male NHANES respondents were included in the final analysis. Participant characteristics are reported as mean ± standard deviation unless otherwise noted. The mean age of the cohort was 37.6 ± 21.8 years. The average dietary creatine intake in this cohort was 0.97 ± 0.87 g/day, while the mean serum concentrations of inhibin B and AMH were 135.7 ± 66.0 pg/mL and 10.45 ± 13.4 ng/mL, respectively. In crude linear regression analysis, creatine intake was not significantly associated with serum inhibin B levels (*B* = −1.49, *P* = 0.48). This relationship remained non-significant after adjusting for age, total caloric intake, and protein intake (*B* = −2.20, *P* = 0.40). Conversely, creatine intake exhibited a significant negative correlation with serum AMH levels in the crude model (*B* = −0.92, *P* = 0.03); however, this association lost statistical significance after adjusting for the aforementioned covariates (*B* = −0.92, *P* = 0.09). Furthermore, no significant differences in creatine intake were observed between participants with inhibin B concentrations below 112 pg/mL and those with values at or above this threshold, which is considered indicative of normal spermatogenesis (Klingmüller & Haidl 1997).

Our findings suggest no significant association between dietary creatine intake and indirect biomarkers of spermatogenesis in males aged 12 years and older. This implies that creatine derived from food sources is unlikely to exert either beneficial or detrimental effects on spermatogenesis in the general population. However, the cross-sectional nature of this study limits the ability to establish causality. Future epidemiological research should investigate diverse cohorts and account for potential environmental and lifestyle factors that may influence spermatogenesis. In addition, interventional studies employing more rigorous methodologies – such as histological assessments of testicular tissue and comprehensive semen analyses across different stages of sperm development – are warranted to provide a clearer understanding of creatine’s role in male reproductive health.

## Declaration of interest

DN, NT, THS and DE declare no known competing financial interests or personal relationships that could have appeared to influence the authorship of this paper. SMO serves as a member of the Scientific Advisory Board on Creatine in Health and Medicine (AlzChem LLC). SMO co-owns patent “*Supplements Based on Liquid Creatine*” at the European Patent Office (WO2019150323 A1) and patent application “*Composition Comprising Creatine for Use in Telomere Lengthening*” at the US Patent and Trademark Office (# 18/934,264). SMO has received research support related to creatine during the past 36 months from the Ministry of Science, Technological Development and Innovation; Provincial Secretariat for Higher Education and Scientific Research; AlzChem GmbH; Kaneka Nutrients; ThermoLife International, and Vireo System Inc. SMO is the co-founder of KRE-ALL, a company developing creatine-enriched food products, and the founder of INOVA Nutrition, a biotechnology startup focused on innovative nutraceuticals.

## Funding

This research did not receive any specific grant from any funding agency in the public, commercial, or not-for-profit sector.

## Author contribution statement

DN, NT, and SMO contributed to conceptualization and investigation. NT and SMO were responsible for methodology. THS and DE helped in supervision. SMO was responsible for writing the original draft. DN, NT, THS, and MAMMF helped in writing review and editing.

## Data availability

All data analyzed are included in the article. Furthermore, inquiries can be directed to the corresponding author.

## Institutional review board statement

The study was conducted according to the guidelines of the Declaration of Helsinki. Ethical approval to conduct the NHANES was granted by the US National Center for Health Statistics Research Ethics Review Board (Protocol #98-12).

## Informed consent statement

Informed consent was obtained from parents or legal guardians of subjects involved in the study.
